# ‘My dad was like “it's your brain, what are you doing?”’: Participant experiences of repetitive transcranial magnetic stimulation treatment in severe enduring anorexia nervosa

**DOI:** 10.1002/erv.2890

**Published:** 2022-02-12

**Authors:** Bethan Dalton, Amelia Austin, Brian C. F. Ching, Rachel Potterton, Jessica McClelland, Savani Bartholdy, Maria Kekic, Iain C. Campbell, Ulrike Schmidt

**Affiliations:** ^1^ Department of Psychological Medicine Section of Eating Disorders Institute of Psychiatry, Psychology & Neuroscience King's College London London UK; ^2^ Department of Psychology Institute of Psychiatry, Psychology & Neuroscience King's College London London UK; ^3^ South London and Maudsley NHS Foundation Trust London UK

**Keywords:** anorexia nervosa, content analysis, eating disorders, qualitative, repetitive transcranial magnetic stimulation (rTMS)

## Abstract

**Objective:**

Repetitive transcranial magnetic stimulation (rTMS) is a promising emerging treatment for anorexia nervosa (AN). However, to date, patients' views and experiences of this treatment have not been fully explored. To assess these, we integrated a qualitative study into a feasibility randomised controlled trial of rTMS in individuals with severe enduring AN.

**Method:**

Twenty‐nine (of 34) trial participants contributed to this study. Semi‐structured interviews were conducted 3‐months following the completion of rTMS treatment (4‐months post‐randomisation), prior to unblinding. Transcripts were analysed using content analysis.

**Results:**

rTMS was deemed an acceptable but time‐consuming treatment. Many emphasised how their lives had changed to some extent during, but mainly after treatment by making them more positive, open‐minded, flexible and willing to try new things in relation to their AN and other aspects of their lives.

**Conclusions:**

These qualitative data will be valuable in shaping participant information, recruitment and planning of future large‐scale trials of rTMS in AN.

**Trial Registration:**

ISRCTN14329415, registered 23rd July 2015, https://www.isrctn.com/ISRCTN14329415

## Highlights

- This study is the first to systematically explore participants' experiences of repetitive transcranial magnetic stimulation (rTMS) treatment for severe enduring anorexia nervosa (AN), as part of a randomised controlled feasibility trial.
- Participants expressed that rTMS was an acceptable but time‐consuming treatment. Many participants who received real rTMS described positive changes in relation to AN and quality of life.
- Findings from this qualitative study will help to shape future research of rTMS treatment for AN.

**Abbreviations**
AN: anorexia nervosa
DBS: deep brain stimulation
DSM‐5: Diagnostic and Statistical Manual of Mental Disorders 5th Edition
ECT: electro‐convulsive therapy
ED: eating disorder
FDA: Food and Drug Administration
NIBS: non‐invasive brain stimulation
NICE: National Institute for Health and Care Excellence
RCT: randomised controlled trial
rTMS: repetitive transcranial magnetic stimulation
SE‐AN: severe enduring anorexia nervosa
TBS: theta‐burst stimulation

## 1 INTRODUCTION

Psychological therapies are the treatment of choice for people with anorexia nervosa (AN), but for adults, outcomes are frustratingly poor (Brockmeyer et al., 2018) with no specific treatment showing superiority (Solmi et al., 2021). Knowledge of the neurocircuitry/neurobiology underlying AN has led to the development of brain‐based/neural models of AN and the call for research on targeted, brain‐directed treatments (Schmidt & Campbell, 2013; Treasure et al., 2020). Non‐invasive brain stimulation (NIBS) techniques are an emerging treatment option for adults with AN (Dalton et al., 2018; Duriez et al., 2020). A particularly promising NIBS technique is repetitive transcranial magnetic stimulation (rTMS), which induces changes in cortical activity in a target brain area that outlasts the duration of stimulation. rTMS has been approved by the US Food and Drug Administration (FDA) and UK National Institute for Health and Care Excellence (NICE) for treating depression and it is under investigation for treating various neuropsychiatric disorders (Brunoni et al., 2019), including eating disorders (EDs) (Duriez et al., 2020). To date, little is known about patients' views and experience of rTMS and other NIBS for any psychiatric disorder (Grycuk et al., 2021; Rosedale et al., 2009), including EDs. In AN, one study assessed 17 patients' hypothetical views of ‘brain interventions’ that might ‘remove’ their illness (Coman, 2017), but given the broad nature of the questions asked, the findings are not particularly informative. In addition, in a case series of 5 AN patients who received 20 sessions of rTMS treatment, informal feedback described improvements in mood, ED symptoms and outlook a few weeks following completion of rTMS treatment (McClelland et al., 2013, 2016).

Ethicists have identified a variety of challenges posed by novel neurotechnologies, such as brain stimulation, in view of these techniques' potential for generating lasting neural, cognitive and behavioural change that may impact on individuals' sense of identity, autonomy and agency. Whilst in many cases these changes may be desired and the main objective of the treatment, some of these changes may be unforeseen, unintended and unwanted (Cabrera et al., 2014). These concerns, both in general and specifically in AN, have mainly been discussed in relation to deep brain stimulation (DBS), but to some extent are also thought to apply to NIBS such as rTMS (Cabrera et al., 2014; Cohen Kadosh et al., 2012; Coman et al., 2014; Illes et al., 2006; Park et al., 2017).

We recently completed a double‐blind feasibility randomised controlled trial (RCT) of 20 sessions of real versus sham high‐frequency rTMS to the dorsolateral prefrontal cortex in 34 adults with severe enduring AN (SE‐AN) (the TIARA study; Bartholdy, et al., 2015; Dalton, Bartholdy, McClelland, et al., 2018). We found that rTMS treatment was feasible, acceptable, safe and well‐tolerated. The clinical outcomes provided preliminary evidence for the therapeutic potential of rTMS in SE‐AN: from baseline (pre‐rTMS) to follow‐up (4‐months post‐randomisation), participants allocated to real rTMS showed large improvements in mood, medium improvements in quality of life and small improvements in body mass index, compared to the sham rTMS group (Dalton, Bartholdy, McClelland, et al., 2018). As part of this trial, in‐depth semi‐structured qualitative interviews were conducted with participants at follow‐up, with the aim of systematically examining participants' views, hopes and concerns regarding rTMS treatment and their experience of receiving real or sham rTMS and participating in the trial.

## 2 METHODS

Ethical approval for the project was obtained from the London – City Road & Hampstead Research Ethics Committee (REC reference: 15/LO/0196).

### 2.1 Participants

Thirty‐four participants with a current Diagnostic and Statistical Manual of Mental Disorders 5th Edition (DSM‐5; American Psychiatric Association, 2013) diagnosis of AN, an illness duration of ≥3 years and at least one previous course of treatment for their ED were enroled in the trial. Following a baseline assessment, participants were randomly allocated to receive real or sham rTMS (17/group). rTMS was administered using the Magstim Rapid device and Magstim D70‐mm air‐cooled real and sham coils. Participants in the real group received 20 sessions of high‐frequency (10 Hz) rTMS at 110% of their individual motor threshold. This consisted of twenty 5 s trains with 55 s inter‐train intervals delivered to the left dorsolateral prefrontal cortex (a total of 1000 pulses delivered over each 20 min session). Sham stimulation was administered at the same parameters using a sham coil. One participant in each group was withdrawn by the researchers prior to starting treatment due to minor safety concerns, and two further participants, both allocated to sham treatment, dropped out during treatment and did not participate in any follow‐up assessments. The remaining 30 participants (16 allocated to real and 14 to sham rTMS) completed the course of rTMS treatment (defined a priori as >17 sessions of rTMS over 4 weeks) and also completed the follow‐up assessment which included the qualitative interview. One participant interview was lost due to technical issues, therefore, data from 29 participants contributed to the present study. Participants' baseline demographic and clinical characteristics are presented in Table 1. All participants provided informed written consent.

**TABLE 1:** Participant characteristics at baseline

	Whole sample (*n* = 29)	Real rTMS (*n* = 15)	Sham rTMS (*n* = 14)
Age (years) (mean ± SD)	30.10 ± 10.78	29.20 ± 9.83	31.07 ± 12.02
AN subtype (AN‐R/AN‐BP) (*n*)	18/11	9/6	9/5
Illness duration (years) (mean ± SD)	15.05 ± 11.33	14.43 ± 11.25	15.71 ± 11.82
Number of previous ED hospitalisations (mean ± SD)	2.31 ± 1.95	2.40 ± 2.16	2.21 ± 1.76
Total duration of previous ED inpatient stays (months) (mean ± SD)	11.56 ± 12.02	12.21 ± 13.28	10.86 ± 10.98

Abbreviations: AN‐BP, anorexia nervosa binge‐eating/purging type; AN‐R, anorexia nervosa restricting type; ED, eating disorders; rTMS, repetitive transcranial magnetic stimulation; SD, standard deviation.

### 2.2 Procedure

The protocol for the TIARA study can be found in Bartholdy, et al., (2015). At the follow‐up assessment (4‐months post‐randomisation), participants completed a questionnaire pack and several computer tasks (data presented elsewhere, Dalton, Bartholdy, McClelland, et al., 2018). Following this, a one‐to‐one in person semi‐structured audio‐recorded interview was conducted by researcher BD (a Research Assistant with a psychology background). BD was involved in the assessment and treatment of participants and therefore, had a prior relationship with them. In addition, for practical reasons, BD was unblinded to the participant’s treatment allocations. The topic guide for the interview was devised for the current study by the authors and is presented in Supplementary A. Participants were asked about their hopes, concerns and expectations regarding rTMS treatment; experience of participation in the trial; observations on effects/side effects of the rTMS; their reports on close others' perspectives on these changes; their views on combining rTMS with psychological treatments for AN; and what they would say to other people considering participation in a similar future trial. Questions were asked in an open manner with as little researcher input as possible. To achieve this, researchers restricted their responses to encouragement, probing and clarification of answers. The interview lasted ≈20 min, and once completed, participants were unblinded to their treatment allocation. It is worth noting here that blinding was successful (as detailed in Dalton, Bartholdy, McClelland, et al., 2018). Audio recordings of the interview were transcribed verbatim and identifying information was removed at point of transcription.

### 2.3 Content analysis

The qualitative data was analysed using inductive content analysis as described by Elo and Kyngäs (2008) using NVivo Version 12. Content analysis is a systematic and objective means of describing and quantifying phenomena and inductive content analysis is used in cases where there is little former knowledge. Each transcribed interview was analysed as a whole and was not limited to the questions asked in the interview. All transcriptions were read multiple times, to gain an overall impression of each participant’s narrative. During this, transcripts were open coded, whereby notes and headings are associated with the text to describe the content. Through a process of abstraction, categories were freely generated based on the codes and were then grouped together in higher‐order categories. Data saturation was achieved at the category level. For each category, a summary of the participants' perspective is provided with supporting quotes, anonymised using participant numbers. Participant numbers prefixed by *R* refer to participants who received real rTMS and those prefixed by *S* refer to participants who received sham rTMS. Frequency counts were calculated and used to describe the proportion of participants mentioning each category. This was split by treatment group (i.e., sham vs. real) where appropriate, for example, when group perspectives differ significantly or when rTMS likely played a role, such as the evaluation of side effects or treatment outcomes. The content analysis was performed by researcher AA (a PhD researcher with a psychology background who had no involvement in the delivery of the trial assessment and interventions and no prior relationship with study participants).

## 3 RESULTS

**FIGURE 1:**
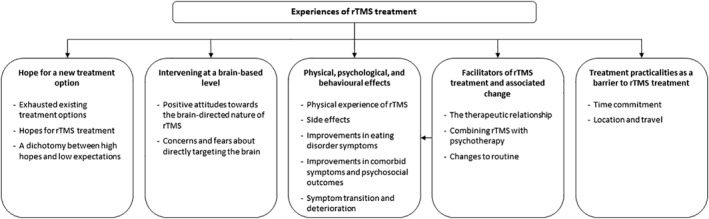
Category map. rTMS, repetitive transcranial magnetic stimulation

Content analysis identified six main categories – hope for a new treatment option; intervening at a brain‐based level; physical, psychological and behavioural effects; facilitators of rTMS and associated changes; treatment practicalities as a barrier – each with their respective sub‐categories. A category map is presented in Figure 1 and the frequency count for each category is shown in Supplementary B (Table S1).

### 3.1 Hope for a new treatment option

Participants described how they had exhausted available treatment options. The opportunity to try a novel treatment provided hope for improvements in AN and related (e.g., depression) symptoms.

*Exhausted existing treatment options.* The enduring nature of illness was a key motivation for pursuing rTMS treatment for most participants (*n* = 24, 84%). Of these, 10 cited previous unsuccessful treatment attempts, and felt that they had exhausted available treatment options. For two, a novel treatment opportunity provided hope after a prolonged period of feeling left behind or forgotten.

*I felt like I had tried a lot of things or everything that was available, and I had these problems for lots of years… other people give up, professionals give up because ‘oh I’ve tried everything’, so when there’s something new it gives you a little hope* S16

*I think that people with long term anorexia are like so ignored, it’s just, in some ways it’s just really nice to know that someone still cares* R22

For a subgroup (*n* = 5), there was an element of desperation, with participants stating that they were willing to try anything to help them recover.

*Hopes for rTMS treatment.* Participants reported a variety of hopes for treatment. Half (*n* = 15, 52%) were altruistically motivated to participate, hoping to contribute to the development of more effective treatments and to help future sufferers.

*I hoped that I might be able to get some benefit from the study, but mostly just that it would in the long‐term lead to better treatments, that was the main thing* S11

*If I can do anything for future sufferers then absolutely sign me up for it, I want to do it* S10

Just over half (*n* = 16, 55%) hoped that rTMS would lead to AN symptom improvement. While this was particularly the case in relation to alleviating ED cognitions and thinking styles (*n* = 10), some expressed ambivalence around potential weight gain following rTMS (*n* = 3).

*…hoping it would change the brain cognition to bring a better outcome and change some habits I want to get rid of* R25

…*it might help me to change some of the ways of thinking… I was hoping that it would matter less about my weight and I’d be able to think about maintaining or gaining… and with the bingeing, needing it less* R4

Three participants added that they wanted to try rTMS treatment with the hope of preventing an inpatient admission.

Three participants hoped that rTMS may help reduce depression symptoms and improve mood. This was of particular importance where participants felt that depression played a role in perpetuating AN, and so improving depression would facilitate ED recovery.

*As I understand it, it seemed to have had some degree of some suggestion it helped mood, so I thought even if it doesn’t help the eating disorder it can help with your mood a little bit* R23

*…with depression and anorexia they kind of go hand in hand so I think even if it works for that [depression], just giving that little bit of a boost to lift somebody will help them with their anorexia and vice versa…* R27

*A dichotomy between high hopes and low expectations.* While eight participants (28%) expressed high hopes for rTMS as a treatment, they reported low expectations for how rTMS would impact them personally.

*Hopeful, but at the same time not actually thinking how could it help… part of me was like what difference can it make…guess I didn’t expect anything, obviously I hoped I’d feel and be different afterwards but I didn’t expect any difference* R13

### 3.2 Intervening at a brain‐based level

Twenty participants (69%) commented on the brain‐directed nature of rTMS. They expressed positive attitudes towards a treatment aimed at the neural/biological basis of AN. However, several participants, as well as family members of some participants, expressed concerns over rTMS intervening with the brain and possible consequences/outcomes.

*Positive attitudes towards the brain directed nature of rTMS.* Several participants (*n* = 5) valued the opportunity to try a novel treatment that focussed on the neural basis of the illness. This included interest in trying something other than talking therapy, a treatment focussed on weight gain, or medication. For two, rTMS fit with their idea of where their illness originated.

*… for me certainly I think cognitive behavioural therapy just isn’t really much of a course, because it all depends on me eradicating things which, I’m afraid, I’ve been rehearsing and practicing for more than 20 years… I welcome anything that penetrates some other way* S6

*A lot of the anorexia treatments are all about feeding you and not really to do with the brain so it’s nice to have something that actually focuses on the fact it’s a mental illness, not something you can just fatten up and then you’re better* R4

One participant felt that the brain‐directed nature of rTMS could reduce stigma around AN.

*It would also validate the disorder more, because there’s still this perception being it’s a vain thing, a diet gone too far, and all those negative stereotypes… it would solidify into people’s heads ‘yes this is actually an illness that needs a form of treatment and now I can see a physical treatment administered as well’* R19

The same participant expressed the belief that rTMS may help streamline treatment pathways for people with comorbidities as rTMS can improve anxiety and depression i.e., rather than going down separate treatment pathways for each condition (AN/anxiety/depression), rTMS might improve all simultaneously.

*Concerns and fears about directly targeting the brain.* People’s reference points for brain‐based treatments tend to be electro‐convulsive therapy (ECT) and how it is portrayed in media. Several (*n* = 7, 24%) expressed that the brain‐directed nature of rTMS was an initial concern. Four participants were initially concerned about possible discomfort or pain associated with the rTMS treatment and one cited worries about losing control of her body in a way reminiscent of ECT (e.g., spasms).

*TMS felt a bit extreme at first because I’m aware I’m doing something actively to my brain* R19

Relatedly, four participants described how their loved ones voiced strong concerns about the action of rTMS during the process of decision‐making around whether to take part in the trial or not.

M*y dad was like ‘it’s your brain, what are you doing’* R27

*I felt perturbed by my sister’s reaction, but I think she was thinking it was deep brain stimulation… some kind of electric shock* S6

One participant also highlighted fears regarding loss of autonomy or agency when receiving rTMS.

*The sort of surrendering control I guess… usually with therapy and stuff you can kind of choose how much you switch on and switch off or whatever but with something that’s going through your brain you don’t have any control over it* S10

Additionally, one participant expressed initial concerns that rTMS could contribute to a loss of identity.

*During the sessions I became less worried about it [rTMS] clinically perverting me as an individual… you weren’t hammering the ‘[participant name]’ out of me* S6

Finally, two participants expressed concern that rTMS treatment could cause deterioration or have ‘reverse effects’ whereby their symptoms would transition from restriction to binge eating.

### 3.3 Physical, psychological and behavioural effects

Participants in both groups described physical sensations associated with rTMS in terms of the actual experience of rTMS and side effects. They also reported beneficial outcomes associated with rTMS, including improvements in ED cognitions and behaviours, mood, cognition, interpersonal relationships, and quality of life. A proportion in the sham treatment group described a deterioration of ED symptoms during or following rTMS treatment (*n* = 5). For the purposes of illustration, quotations for this theme will be limited to those participants who received real rTMS.

*Physical experience of rTMS.* Twelve participants (real *n* = 8, sham *n* = 4) commented on the physical sensation experienced during the rTMS. Of these who received real rTMS, they described the sensation as strange and weird, with most describing it as not painful. Three of these reported that this was particularly the case in the first session, but they got used to the sensations over the duration of treatment.

*… it wasn’t necessarily discomfort, it just felt odd to begin with, I just got used to it, it didn’t hurt or anything* R7

*Side effects.* Eleven participants in each group attributed a range of side effects to the rTMS treatment (real = 73%, sham = 79%). This included pain or headache (real *n* = 10, sham *n* = 10), tiredness/exhaustion (real *n* = 4, sham *n* = 3), and nausea (real *n* = 2, sham *n* = 1). Of those reporting side effects attributed to rTMS, 10/11 (91%) participants in the sham group and 7/11 (64%) in the real group reported that these effects improved as the treatment progressed. One participant suggested that guidance on how to manage side‐effects would be helpful.

*Improvements in eating disorder symptoms.* Underlying ED cognitions were reported to improve in 12 participants (real *n* = 8, sham *n* = 4). This included a reduced need for control over food (real *n* = 4, sham *n* = 0), increased awareness of having an ED (real *n* = 1, sham *n* = 1), reduced guilt associated with eating (real *n* = 1, sham *n* = 0), and increased motivation and determination to recover (real *n* = 5, sham *n* = 2).

…*I’m not as anxious around food. I know before if something got put in front of me and it was a fear food or something, I would literally cry and be really panicky and I couldn’t cope with it whereas as now I feel calmer and I just almost look at it like okay this is difficult but I’ve got to do it, it’s like more acceptance* R7

*I just got increasingly more relaxed as I kinda went on, kind of general but more specifically with food as well, like later on in the weeks when I came home, my parents cooked me tea, which I never normally did* R2

Fourteen participants reported positive changes in their eating behaviours during or following the trial (real *n* = 9, sham *n* = 5). This included less food restriction (e.g., variety, amount; real *n* = 7, sham *n* = 3), less binge eating (real *n* = 1, sham *n* = 1), and less compensatory exercise (real *n* = 2, sham *n* = 2). Five (real *n* = 4, sham *n* = 1) reported weight gain since the beginning of treatment.

*I was a lot freer with food kind of made choices… and things like if there were four chocolates left in a packet and my snack was only three, having the kind of rational thinking ‘what’s the point of leaving one and I’ll just have it’, whereas before I would have been ‘well there is four so if I just have two now I can have two tomorrow’ and I would have cut down my snack then so very very subtle but big* R27

… *recently in the last few weeks,* [I] *eat normally and eat whatever I want to* R13

*Improvements in comorbid symptoms and psychosocial outcomes.* Participants described positive changes in relation to their mood and quality of life. Thirteen (real *n* = 9, sham *n* = 4) reported experiencing heightened positive mood and a significant reduction in depression symptoms during the rTMS treatment or the follow‐up period.

*Right about the second week, I’ve for years woken up feeling really depressed, and didn’t want to get out of bed. I’ve always gotten out of bed, but I’ve had this fog and this fog started to lift. Since that second week, I haven’t woken up feeling depressed, which is a huge change* R9

*I did feel like my mood had improved… I definitely didn’t feel as agitated or kind of touchy about things* R12

Two participants in the real group, however, reported that the positive impact on mood was short‐lived but still appreciated the temporary improvement.

*…if like the upswing in mood was due to like the TMS, then even if it hasn’t really lasted, it was still like amazing to feel different for a little bit* R22

Fourteen participants (real *n* = 9, sham *n* = 5) reported improvements in cognitive functioning. This included increased mental flexibility and decreased rigidity in both groups (real *n* = 5, sham *n* = 5) and, only in the real group, improved concentration (*n* = 2).

*everything is less black and white I think… so in terms of exercise, before I could be really tired and I didn’t want to do it but I felt like that wasn’t a choice, I had to do it, I had to exercise, whereas now I can sort of think ‘I don’t have to’ and ‘that’s not the right thing to do’, and think a bit more rationally* R7

*I’m more relaxed so if something goes wrong and plans change, I can do that quite easily* R29

Ten participants (real *n* = 9, sham *n* = 1) reported improved interpersonal connections. In the real group, this included stronger relationships with friends and family (*n* = 5) and a fuller social life (*n* = 6). The improvements in relationships and socialising in the real rTMS group were underpinned for two participants by increased assertiveness and for one participant, a decrease in reactivity.

*I’m being able to put people before the eating disorder. That’s been the biggest change since having the treatment* R3

*… it’s made me more assertive, more flexible, just more accepting to change and able to go with the flow, more spontaneous, leave the rigid ‘I will not move out of this structure no matter what happens’, which has abled me to open up new relationships, old relationships, friends* R25

Seven participants (real *n* = 6, sham *n* = 1) reported that they felt a greater sense of identity and four participants in the real group expressed improved self‐esteem and confidence.

*Before the TMS I felt rotten, and now I feel like I’m sort of getting back to my old confident self, who’s beginning to trust myself again* R9

Nine participants (real *n* = 8, sham *n* = 1) suggested that the trial had positive impacts on functional aspects of their everyday lives. In the real group, this included return to or improved experience of work or university (*n* = 4) and going back to or finding new interests and hobbies (*n* = 2). These functional improvements outside the home were often underpinned by an increased feeling of independence (*n* = 4). Relatedly, two participants from the real rTMS group reported improvements in their sleep.

*I’ve found it easier to be motivated to do things and like make small changes… I’ve started* [horse] *riding and… I managed to organise it and stuff like that so, like socially things and fun things, I’ve had more motivation to do them and like organise stuff for them, and I’m working more regularly* R4

[I’m] *a lot more independent… able to kind of fend for myself, a bit more proactive, with like going out and getting my job… I’m a lot more thinking about the future rather than, before I just kind of didn’t give it any thought, I was like ‘Well I’m not bothered what happens anyway’ whereas now its like applying to uni again and making sure it’s the course I wanna do and things like that* R2

*Symptom transition and deterioration.* One participant in each group experienced a symptom transition, with the participant in the real rTMS group developing binge eating behaviours and the one in the sham group exchanging vomiting for compulsive exercise.

About one third of the participants in the sham group (5/14, 36%) and one participant in the real group reported a general deterioration during the rTMS treatment or the follow‐up period.

*I don’t know it felt a bit like just probably felt like it got worse for me, but I don’t know if that’s just because I was here or it was just getting worse anyways…*S24

### 3.4 Facilitators of repetitive transcranial magnetic stimulation treatment and associated change

Participants identified several factors that they felt facilitated or are potential facilitators of rTMS treatment engagement and beneficial outcomes, including the role of the rTMS therapist, using rTMS as an adjunct to psychotherapy, and changes to their pre‐existing routines.

*The therapeutic relationship.* The majority (*n* = 27, 93%) reported that the role of the rTMS therapist was important to treatment. Several (*n* = 13) appreciated that the researcher put them at ease, reassured them, and reduced their anxieties about treatment. Seventeen participants commented that interacting with the rTMS therapist gave them a chance to socialise and reduced the isolation characteristic of AN. Four added further insight, suggesting that these positive interactions with and support from the rTMS therapist improved attendance.

*You were my social life, I didn’t have one otherwise and people with eating disorders are often very isolated so having a chat was sort of quite nice* S1

*Combining rTMS with psychotherapy.* Half of all participants (*n* = 15; real *n* = 9, sham *n* = 6) suggested rTMS would work best if accompanied by psychological therapy. For 10 participants, this was because they felt that, if rTMS resulted in changes, it would be useful to talk these through to better understand them and know how to cope with them. For eight participants, it was thought that a clinician would be well placed to help a patient notice rTMS‐related changes and capitalise on increased motivation and early cognitive change, which may be beneficial in promoting a better mental space to engage in therapy.

A*s the kind of changes taking place in your brain and you’ve got somebody to help you kind of understand that and cope with it because I found when I did become a bit freer with food it was a bit scary… like it was ‘oh my goodness I’m actually eating this' so it would have been good to have had somebody to work through that with…* R27

*It* [rTMS] *might put you in a head space to kind of be more open to receiving other kinds of therapy… if it makes you a bit more relaxed and open, you might be more open minded to take on what other people are saying to you rather than shrugging it off straight away* R2

Six participants highlighted that clinicians need to be aware of overloading the patient, particularly given the time involved in rTMS treatment in combination with other treatment and life commitments. One emphasised the importance of having talking therapy for AN before trying rTMS and felt that rTMS treatment may be more appropriate for people with a longer illness duration.

*Changes to routine.* Three participants expressed that changes to their routine due to the structure of rTMS treatment contributed to the beneficial outcomes they experienced during the trial. For one, attending daily rTMS treatment sessions encouraged a better routine, which led to reduced binge eating and contributed to feeling more in control and better about themself. For the others, a release from everyday routines was associated with positive outcomes.

*It was also good to just take myself away from of the routines that I normally place myself in which was work at the time and home routine, it was really nice to kind of be away from all of that and be in a different setting* R12

### 3.5 Treatment practicalities as a barrier to repetitive transcranial magnetic stimulation treatment

More than half of participants (*n* = 17, 59%) were concerned about the practicalities of treatment prior to taking part. Following treatment, thirteen (45%) identified time and location related practicalities as barriers.

*Time commitment.* For 10 participants, the time commitment associated with the treatment and subsequent absence from education or work was an initial concern. Post‐treatment, this was identified as a barrier by seven participants. For four, the demanding treatment schedule conflicted with their ED symptoms.

*It’s a lot more than any other treatments I have experienced* R23

*… the fact I was coming up every day probably made me a bit worse in terms of I was skipping lunch and stuff* R7

Suggestions for a more feasible treatment protocol (length and frequency of sessions, *n* = 4) were made, including reduced weekly frequency but over a longer period (e.g., 2–3 months), reduction of the inter‐train interval time, and several treatments in one day for a shorter overall treatment period.

*Location and travel.* Eight participants were initially concerned about travelling to and from appointments, with this worry sometimes underpinned by concerns that ED symptoms could get in the way (*n* = 2).

*…my stamina has decreased, so getting back into commuting, that physical exertion was a little bit intimidating* R19

Indeed, for 10 participants, the location and related travel was cited as a barrier to treatment. For a few participants, this meant relocating for the duration of the treatment and being away from support networks. Four suggested that increasing the number of locations across the country should be considered and that rTMS should be delivered in the same location as other treatments.

## 4 DISCUSSION

As part of a feasibility RCT, we explored the treatment experience of rTMS in people with SE‐AN. The data highlight a demand for novel treatments in this patient group: participants were hopeful for improvements in their symptoms and keen to take steps to recover but had struggled to effect change with the available treatments, in combination with the severe and enduring nature of their illness. rTMS was seen to be an acceptable treatment option, with 67% of the people who received real rTMS reporting they would recommend it to others with AN. Participants identified facilitators and barriers of rTMS treatment which will be important to address in future rTMS trials.

The brain‐directed nature of rTMS was popular among participants as they felt that the treatment reflected what they believed about AN being a brain‐based disorder. Furthermore, the biological focus gave credibility to this treatment and for one participant, they felt a biological treatment would help reduce stigmatising attitudes towards AN. Two participants reported having initially had concerns about potentially losing their identity or their autonomy or agency, in that they felt that, unlike in psychotherapy, with rTMS they had no control over their level of engagement in the treatment. Outside of the present data, at screening, one trial participant raised concerns about possible negative effects of rTMS on their intelligence. However, these concerns did not persist when people participated in the treatment. Our findings suggest that some of the concerns raised by ethicists are not borne out by the data. However, we did not specifically ask about perceived ethical issues. Of note, in a qualitative study of the experience of transcranial direct current stimulation in combination with approach bias modification training in participants with binge eating disorder (Gordon et al., 2021), autonomy was identified as a pertinent issue for a minority of participants, with one feeling the intervention was autonomy‐enhancing and another autonomy‐compromising. Therefore, future qualitative studies may benefit from systematically exploring the ethical implications of rTMS treatment for AN.

Many participants who received the real rTMS treatment emphasised how their lives had changed to some extent during, but mainly after treatment, such that they felt more positive, open‐minded, motivated, flexible and willing to try new things, both in relation to their ED and other aspects of their lives. The longer‐term persistence of these changes was reflected in written feedback at the 18 months post‐randomisation open follow‐up (Dalton, Lewis, et al., 2020). Several participants who received real rTMS reported a reduced anxiety around food and a tendency to be more flexible around food and eating (e.g., eating a greater variety of foods). These specific improvements were not reflected when assessed by questionnaire (Dalton, Bartholdy, McClelland, et al., 2018); however, this increased flexibility in food choice was seen in findings on the Food Choice Task where participants showed a decrease in self‐controlled food choices following real rTMS (Dalton, Foerde, et al., 2020). These qualitative data also provide potential context to our neuroimaging findings (Dalton et al., 2021), in which we proposed that the observed reduction in amygdala activity (over the rTMS treatment period) may be associated with longer‐term weight gain due to an improved ability to tolerate uncomfortable physical and emotional sensations (e.g., anxiety or fear around food). It is of interest that several participants who received sham rTMS also reported positive changes during and following rTMS treatment. Indeed, placebo effects associated with sham rTMS procedures have been documented (Burke et al., 2019). Taken together, these qualitative data add relevant context to the quantitative data, highlighting the value and importance of these experienced changes for the individual, even if seemingly subtle.

In SE‐AN, depression has been associated with a poor quality of life (Arkell & Robinson, 2008) and reducing these symptoms is often emphasised in management strategies for this patient group (Wonderlich et al., 2012). The largest treatment effects in the trial were seen in relation to mood, with moderate effects on quality of life (Dalton, Bartholdy, McClelland, et al., 2018). Findings from the present study mirrored the quantitative improvements in mood and quality of life following real rTMS treatment. Improved relationships with significant others, greater engagement in social activities and hobbies, and increased confidence and self‐esteem were all seen to contribute to an overall better quality of life.

Patients identified key facilitators of rTMS treatment: the role of the rTMS therapist and combining rTMS with psychotherapy. Rapport with the rTMS therapist appeared to influence treatment experience and facilitate attendance and treatment completion. Connection with the rTMS therapist was similarly identified as an important aspect of rTMS treatment for patients undergoing rTMS for depression (Rosedale et al., 2009). Secondly, it was felt that engagement in talking therapies alongside rTMS treatment would have been a beneficial source of support and an opportunity to facilitate and bolster any rTMS‐related changes. It was also suggested that rTMS may help to put individuals in a more appropriate ‘head‐space’ to engage with talking therapies. Indeed, it has been proposed that brain stimulation treatments will be most effective as an adjunct to psychological therapy and/or cognitive training (Bajbouj & Padberg, 2014; Elmasry et al., 2015; Tsagaris et al., 2016). Preliminary support has been provided for this in individuals with major depressive disorder undergoing rTMS combined with psychotherapy (Donse et al., 2018). However, participants in the present study felt that consideration needed to be paid to the demanding nature of the rTMS treatment protocol when using rTMS as an adjunct to existing evidence‐based psychotherapies.

Practicalities involved in the rTMS treatment were identified by most participants as a barrier: it required attending the TMS clinic on consecutive weekdays for 4 weeks. Participants found this substantial commitment physically demanding and time‐consuming. Indeed, some reported associated deterioration in their ED. In depression, other forms of rTMS with less time‐intensive protocols (i.e., shorter and fewer sessions and the option of multiple sessions in one day) have been shown to achieve comparable effects to standard rTMS protocols (as used here) (Schwippel et al., 2019). For example, theta‐burst stimulation protocols are less time intensive and are currently being researched in SE‐AN (Gallop, 2019). If effective, these will significantly reduce the burden on participants.

### 4.1 Strengths and limitations

This is the first study to systematically collect qualitative information about participants' experience of rTMS treatment in the context of an RCT. These qualitative data provide a rich source of information that will be valuable in shaping participant information, guiding recruitment and planning other aspects of the study design of future larger‐scale trials in this area.

There are some limitations. Participants were asked to reflect over the whole period from getting involved in the study to the 4‐months post‐randomisation follow‐up. Therefore, there may be some recall bias in participants' retrospective recollections of treatment. It may be useful in future to perform regular qualitative assessments throughout treatment to develop a more thorough understanding of patient experience (e.g., use of a diary). Lastly, interviews were performed by an unblinded researcher involved in the delivery of research assessments and rTMS treatment. This was for practical capacity reasons within the research team.

### 4.2 Conclusions

Incorporating qualitative data collection in feasibility trials provides a valuable opportunity for evaluating the trial from the patient perspective and allows for further elaboration outside of the gathered quantitative information (Crawford et al., 2002). Participants provided insights into their reasons for pursuing a new treatment, their hopes and expectations, the practicalities of trial involvement, and recommendations for future research protocols. The findings also add context to the quantitative findings, particularly in relation to the beneficial outcomes experienced by some participants. The information will be of use for future brain stimulation trials in this patient group, including informing the planning of the study design (e.g., rTMS protocol, accessibility) of future larger‐scale trials and improving patients' experience of undergoing rTMS.

## CONFLICT OF INTEREST

The authors declare that they have no conflict of interest.

## Supporting information

Supporting Information

## Data Availability

The data that support the findings of this study are available from the corresponding author upon reasonable request.
