# Supplementary material for: ‘My dad was like “it's your brain, what are you doing?”’: Participant experiences of repetitive transcranial magnetic stimulation treatment in severe enduring anorexia nervosa
Source: Eur Eat Disord Rev. 2022 Feb 12;30(3):237–49. doi: 10.1002/erv.2890 (PMC9304183; doi:10.1002/erv.2890)
Supplement: Supplementary file 1 — Supporting Information [file ERV-30-237-s001.docx]

**“My dad was like ‘it’s your brain, what are you doing?’”: Participant experiences of repetitive transcranial magnetic stimulation treatment in severe enduring anorexia nervosa**

**Supplementary A:** Topic guide for patient interview

If you cast your mind back to before you started the study: Why did you want to take part in the study? How did you feel about taking part?

What were your hopes and expectations before starting the sessions? Was there anything you were looking forward to, was there anything you were not looking forward to? If so, what were they?

What was your experience of the treatment? Did that change over the course of the sessions? If so, in what way?

PROMPT: Were there factors that supported or got in the way of the treatment?

Did you have any other treatment alongside the study e.g. psychological therapy?

PROMPT: If yes, how did you feel rTMS worked alongside your other treatment?

PROMPT: If no, how do you think rTMS would work alongside other treatments?

How important was the role of the TMS therapist?

Have you noticed any changes during or as a result of rTMS treatment? For example, has your thinking, feelings or who you are as a person changed during the course of these sessions

Do other people think there have been changes, things that are different now, ways in which you are different since having the rTMS?

Thinking back to your hopes and expectations about the study, did you get out of the treatment what you hoped you would? If any, what benefits would you say you have found with this treatment? Did you experience any negative outcomes as a result? If, so what were they?

Overall, what do you think about rTMS as a treatment option for anorexia and other eating disorders?

What would you tell others thinking of taking part in rTMS treatment?

Do you have any recommendations about the treatment or about participating in this study or any ideas on how the treatment process could be improved?

Is there anything else you would like to say about the treatment or about participating in this study?

**Supplementary B:** Table S1. Coding framework constructed from the data using inductive content analysis with frequency counts for whole sample (n=29), and additionally separated out for real (n=15) and sham (n=14) rTMS groups on relevant categories.

| **Main category** | **Generic categories with sub-categories** | **Real (*n)*** | **Sham (*n)*** | **All (*n)*** |
| --- | --- | --- | --- | --- |
| **Hope for a new treatment option** | Exhausted existing treatment options | | | |
|  | Enduring Illness |  |  | 24 |
|  | Previous unsuccessful treatment attempts |  |  | 10 |
|  | Feeling forgotten |  |  | 2 |
|  | Desperation |  |  | 5 |
|  | Hopes for rTMS treatment | | | |
|  | Altruism |  |  | 15 |
|  | Improvement in AN symptoms |  |  | 16 |
|  | Prevent an inpatient admission |  |  | 3 |
|  | Improvement in mood |  |  | 3 |
|  | A dichotomy between high hopes and low expectations | | | |
|  | A dichotomy between high hopes and low expectations |  |  | 8 |
|  | | | | |
| **Intervening at a brain-based level** | Positive attitudes towards the brain-directed nature of rTMS | | | |
|  | Novel neurological-focused approach |  |  | 5 |
|  | Fit with personal beliefs of illness origin |  |  | 2 |
|  | Reduce stigma |  |  | 1 |
|  | Streamline treatment for comorbidities |  |  | 1 |
|  | Concerns and fears about directly targeting the brain | | | |
|  | Personal concerns |  |  | 7 |
|  | Anticipation of physical sensations |  |  | 5 |
|  | Concerned loved ones |  |  | 4 |
|  | Loss of autonomy / agency |  |  | 1 |
|  | Loss of identity |  |  | 1 |
|  | Deterioration or ‘reverse effects’ |  |  | 2 |
|  | | | | |
| **Physical, psychological, and behavioural effects** | Physical experience of rTMS | | | |
|  | Physical experience of rTMS | 8 | 4 | 12 |
|  | Side effects | | | |
|  | Side effects | 11 | 11 | 22 |
|  | Pain/headache | 10 | 10 | 20 |
|  | Tiredness | 4 | 3 | 7 |
|  | Nausea | 2 | 1 | 3 |
|  | Improved with time | 7 | 10 | 17 |
|  | Improvements in eating disorder symptoms | | | |
|  | Eating disorder cognitions | 8 | 4 | 12 |
|  | Less need for control of food | 4 | - | 4 |
|  | More awareness of eating disorder | 1 | 1 | 2 |
|  | Less guilt associated with eating | 1 | - | 1 |
|  | Increased motivation and determination to recover | 5 | 2 | 7 |
|  | Eating disorder behaviours | 9 | 5 | 14 |
|  | Less food restriction | 7 | 3 | 10 |
|  | Less binge eating | 1 | 1 | 2 |
|  | Less compensatory exercise | 2 | 2 | 4 |
|  | Weight gain | 4 | 1 | 5 |
|  | Improvements in comorbid symptoms and psychosocial outcomes | | | |
|  | Improved mood | 9 | 4 | 13 |
|  | Short-lived mood improvement | 2 | - | 2 |
|  | Improvements in cognitive functioning | 9 | 5 | 14 |
|  | Increased mental flexibility / decreased rigidity | 5 | 5 | 10 |
|  | Improved concentration | 2 | - | 2 |
|  | Improved interpersonal connections | 9 | 1 | 10 |
|  | Stronger relationships | 5 | - | 6 |
|  | Fuller social life | 6 | - | 5 |
|  | Greater sense of identity | 6 | 1 | 7 |
|  | Improved self-esteem / confidence | 4 | - | 4 |
|  | Positive impact on daily function | 8 | 1 | 9 |
|  | Increased feeling of independence | 4 | - | 4 |
|  | Improved sleep | 2 | - | 2 |
|  | Symptom transition and deterioration | | | |
|  | Symptom transition | 1 | 1 | 2 |
|  | Deterioration | 1 | 5 | 6 |
|  | | | | |
| **Facilitators of rTMS treatment and associated change** | The therapeutic relationship | | | |
|  | Important role of rTMS therapist |  |  | 27 |
|  | Reduced anxiety |  |  | 13 |
|  | Opportunity for socialising |  |  | 17 |
|  | Improved attendance |  |  | 4 |
|  | Combining rTMS with psychotherapy | | | |
|  | Positive attitude / recommended |  |  | 15 |
|  | Support in coping with changes |  |  | 10 |
|  | Capitalising on increased motivation and rTMS-related change |  |  | 8 |
|  | Recommend awareness of possible overload |  |  | 6 |
|  | Changes to routine | | | |
|  | Changes to routine |  |  | 3 |
|  | Encouraged a better routine |  |  | 1 |
|  | Release for everyday routine |  |  | 2 |
|  | | | | |
| **Treatment practicalities as a barrier to rTMS treatment** | Time commitment | | | |
|  | Initial concerns |  |  | 10 |
|  | Barrier to treatment |  |  | 7 |
|  | Conflicted with eating disorder |  |  | 4 |
|  | More feasible treatment protocol recommended |  |  | 4 |
|  | Location and travel | | | |
|  | Initial concerns |  |  | 8 |
|  | Barrier to treatment |  |  | 10 |
|  | Multiple locations recommended |  |  | 4 |
